# In-Hospital Mortality Among Elderly Patients Hospitalized for Femur Fracture with and Without Diabetes Mellitus: A Multicenter Case–Control Study

**DOI:** 10.3390/jcm13216484

**Published:** 2024-10-29

**Authors:** Lavinia Jürgens, Theresia Sarabhai, Karel Kostev

**Affiliations:** 1 Unfallchirurgie und Orthopädie, Klinikum Neukölln, 12351 Berlin, Germany; 2 Department of Endocrinology and Diabetology, Medical Faculty and University Hospital Düsseldorf, Heinrich-Heine-University, 40225 Düsseldorf, Germany; 3 University Hospital, Philipps-University, 35043 Marburg, Germany

**Keywords:** diabetes mellitus, hip fracture, mortality, risk factor, elderly, Germany

## Abstract

**Purpose**: The aim of the present study was to explore whether diabetes mellitus (DM) is associated with in-hospital mortality in patients admitted for femur fractures. **Methods**: Our multicenter case–control study included patients aged ≥65 with a primary diagnosis of femur fracture with and without DM treated in 36 German hospitals between January 2019 and December 2023. Patients with DM were matched to patients without DM (1:3) using propensity scores based on age and sex. Multivariable logistic regression analyses were conducted to assess the associations between DM and in-hospital mortality. **Results**: A total of 3220 patients with diabetes and 9660 patients without diabetes were included (mean age: 83 years). The overall in-hospital mortality rate was higher in DM patients (6.4%) than in those without DM (5.4%). However, the association between DM and in-hospital mortality was not significant after adjustment for other co-diagnoses. In particular, atrial fibrillation, heart failure, and CKD attenuated the effect of DM on in-hospital mortality. **Conclusions**: Our data show that DM is not significantly associated with in-hospital mortality in femur fracture patients. However, the presence of other comorbidities may influence mortality outcomes, highlighting the need for early intervention and targeted treatment to improve patient outcomes.

## 1. Introduction

Skeletal fractures represent a major public health concern given their high incidence and prevalence, particularly within the elderly population. The Global Burden of Disease Study reported a 30.3% increase in fractures between 1998 and 2019, with older individuals being at higher risk [1]. Germany reported an increase of 14% from 2009 to 2019 and a lifetime prevalence of 44% in adults aged 55 years and older [2]. Fragility fractures, which are associated with low-impact trauma and osteoporosis, are the most common fracture type among older people. Although the age-specific incidence of fragility fractures varies demographically, one in three women and one in five men will experience such a fracture in their lifetime [3]. As the world’s population ages, the incidence of these fractures is expected to increase, with the femur, vertebrae, and wrist being the most common sites [4]. Thus, femur fractures represent a major public health concern and are associated with increased hospitalization, morbidity, and mortality rates [5].

Femur fractures typically necessitate surgical intervention, such as osteosynthesis, total hip arthroplasty, or hemiarthroplasty. These procedures carry an increased mortality risk due to a range of severe complications, including blood loss, various tissue infections, and cardiovascular incidents such as heart attack or stroke. Furthermore, the immobilization and prolonged hospitalization required for fracture management increase the likelihood of developing pneumonia, deep vein thrombosis, and consequently, pulmonary embolism [6]. Numerous studies have linked hip or femur fractures to elevated in-hospital morbidity and mortality rates [7,8,9,10,11]. For instance, Groff et al. report an overall in-hospital mortality rate of 3% in patients undergoing surgery after hip fracture [10], and Rapp et al. report a mortality rate of 10–15% within the first 30 days after hip surgery [11].

The higher mortality rates associated with hip and femur fractures are predominantly observed in elderly patients, who often have a high prevalence of comorbidities [12,13].

Diabetes mellitus (DM) is the eighth leading cause of mortality globally [14] and is increasingly prevalent in the elderly population in Europe, with an estimated prevalence of 33% in adults aged 65 or above, who are primarily affected by type 2 diabetes mellitus (DM2) [15]. Further chronic diseases including dyslipidemia, hypertension, and central obesity may precede or accompany DM. When these diseases occur together, they strongly increase the risk of cardiovascular complications and mortality [16]. Furthermore, DM and osteoporosis seem to have a bi-directional relationship [17], where altered glucose metabolism is linked to increased osteoclast activity and reduced osteoblast formation, resulting in diminished bone strength and a higher risk of fracture [18,19,20]. Therefore, both type 1 diabetes mellitus (DM1) and type 2 diabetes mellitus (DM2) are associated with an increased risk of femur fracture, as described in numerous previous studies [21,22,23].

Few previous studies have directly examined the effect of DM on in-hospital mortality in an older population over 65 years of age. A retrospective cohort study in Sydney involving 139,321 people found that DM did not impact inpatient mortality in the elderly cohort [24]. Conversely, Schmitt et al. found that DM increased the risk of adverse events such as pulmonary embolism in hospitalized patients [25], although their study did not account for patient age or specific admission for femur fracture. While the overall mortality rate among patients with DM is well documented, there is still considerable uncertainty regarding the impact of DM on in-hospital mortality for specific conditions such as femoral fractures in older patients. Previous research indicates that individuals with DM and femoral fractures face increased risks of postoperative complications, prolonged recovery periods, and hospital stays [26,27]. However, the existing literature regarding the direct contribution of DM to increased in-hospital mortality among older patients with femoral fractures is inconclusive.

Therefore, the aim of this study is to investigate the impact of DM on the in-hospital mortality rate of patients admitted primarily with femur fracture. By gaining a better understanding of the potential association between DM and an elevated risk of in-hospital mortality following a femur fracture, healthcare professionals can identify those at risk and promptly implement optimal treatment strategies.

## 2. Materials and Methods

### 2.1. Data Source

This multicenter case–control study was based on data reported to the hospital database (company: IQVIA, Frankfurt am Main, Germany) by the Institute for Hospital Reimbursement (InEK) under Section 21 of the Hospital Fees Act (KHEntgG), which contains data from 36 hospitals across Germany, including specialized, primary care, maximum care, standard care, and university hospitals [28,29]. The individual treatment episodes per case (Section 21 dataset) were grouped using specialized grouping software developed jointly by 3M Health Information Systems and IQVIA. All export files generated by the software (e.g., case and patient number) were anonymized before transmission for data protection reasons.

### 2.2. Study Population

This multicenter cross-sectional study included all hospitalized patients aged ≥65 admitted between January 2019 and December 2023 with a primary diagnosis of femur fracture (ICD-10: S72). Patients were classified as with and without diabetes mellitus (ICD-10: E10–E14) based on their secondary diagnoses. In compliance with European and German national legislation, no individual consent was required for inclusion in this study.

### 2.3. Study Outcome and Covariables

The outcome of this study was an analysis of the prevalence of in-hospital mortality after femur fracture in elderly patients with and without diabetes mellitus. The dataset used includes death as one of the discharge types. The proportion of patients who died in hospital was calculated separately for women and men and was also stratified by age group (65–69, 70–79, 80–89, 90+ years).

### 2.4. Statistical Analyses

To avoid selection bias and to adjust for the effect of age and sex on outcomes, patients with diabetes were matched to patients without diabetes (1:3) using nearest neighbor propensity scores based on age and sex. The standardized mean difference (SMD) was used to examine the balance of the covariate distribution between matched pairs. An SMD of less than 0.1 was accepted as an indication of adequate covariate balance. The differences in comorbidities between patients with and without DM were compared using Chi-squared tests.

Multivariable logistic regression analyses were performed to assess the associations between DM and in-hospital mortality. The multivariable regression analyses were adjusted for the most common diagnoses, including hypertension [ICD-10: I10], dyslipidemia [ICD-10: E78), chronic ischemic heart disease [ICD-10: I25], atrial fibrillation and flutter [ICD-10: I48], heart failure [ICD-10: I50], chronic kidney disease [ICD-10: N18], and all-cause dementia [ICD-10: F00–F03, G30]. Analyses were performed for the total population and for 6 age groups (65–69, 70–79, 80–89, and 90+ years), as well as separately for female and male patients.

Furthermore, the impact of each co-diagnosis on the association between DM and in-hospital mortality for older patients with femur fracture was investigated in a separate analysis. The model included diabetes as a dependent variable, and each further diagnosis was then added as an independent variable until all variables were included.

The results of the logistic regression models were presented as the odds ratio (OR) with 95% Confidence Intervals (CIs). *p*-values < 0.05 were considered statistically significant. All analyses were performed using SAS 9.4 (SAS Institute, Cary, NC, USA).

## 3. Results

### 3.1. Baseline Characteristics

**Figure 1:**
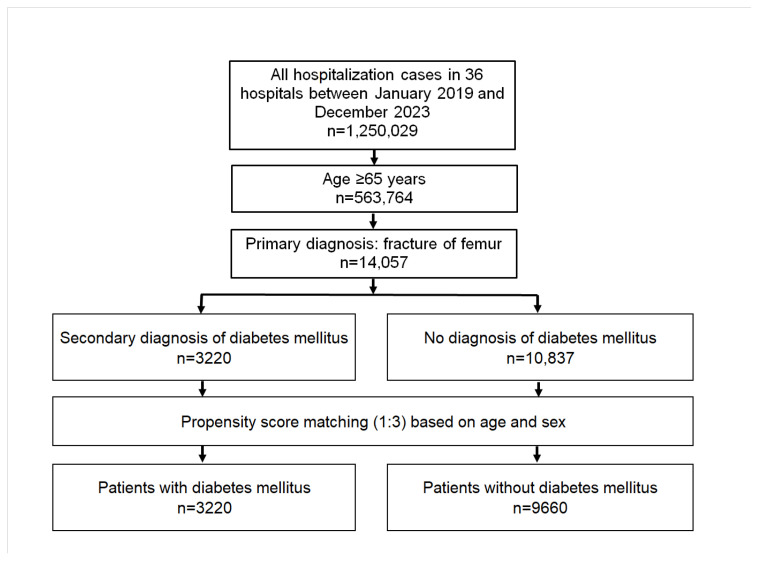
Flow chart of the selection process for hospitalized elderly people with femur fracture with or without diabetes mellitus.

After the matching process, we identified and included 3220 patients with a known DM diagnosis and 9660 patients without diabetes (Figure 1). The mean age for both groups was ~83 years, with ~72% of participants aged 80 years or older (Table 1). In both groups, 69.1% of patients were women (Table 1). The most prevalent type of fracture was the fracture of the head and neck of the femur (ICD-10: S72.0, 46%), followed by pertrochanteric fracture (ICD-10: S72.1, 38%). Other types of fractures were subtrochanteric fracture (ICD-10: S72.2, 7%), fracture of the shaft of the femur (ICD-10: S72.3, 6%), and fracture of the lower end of the femur (ICD-10: S72.4, 3%).

The proportion of co-diagnoses, especially hypertension, dyslipidemia, chronic kidney disease, and heart failure, was higher among patients with DM than among those without DM (Table 1). Interestingly, only the comorbidity all-cause dementia did not differ between the groups (Table 1). The length of hospital stay (LOS) was 17.4 (standard deviation, SD: 10.5) days in DM and 16.8 (SD: 10.0) days in non-DM patients.

### 3.2. Prevalence of In-Hospital Mortality in Older People With and Without Diabetes Mellitus After a Femur Fracture

**Figure 2:**
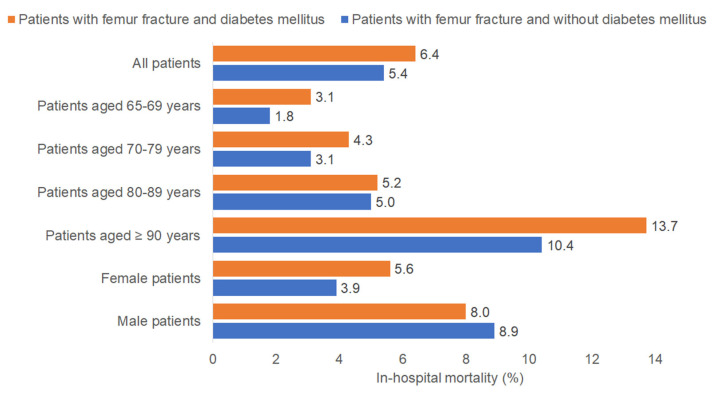
In-hospital mortality rate in femur fracture patients with and without diabetes mellitus.

Figure 2 shows the rate of in-hospital mortality in older patients with femur fracture with and without DM. The overall in-hospital mortality rate was higher in patients with DM (6.4%) than in those without DM (5.4%). The in-hospital mortality after femur fracture increased with age. In patients aged 65–69 years, the in-hospital mortality after femur fracture was 3.1% for patients with DM and 1.8% for those without DM. In patients aged 70–79 years, the in-hospital mortality after femur fracture was 4.3% for patients with DM and 3.1% for those without DM. In patients aged 80–89 years, the in-hospital mortality rates were similar for DM (5.2%) and non-DM (5.0%) patients. The highest rate of in-hospital mortality was found in patients over 90 years of age, who also showed the largest difference between the groups (13.7% with DM vs. 10.4% without DM). Female patients had a higher in-hospital mortality rate after femur fracture among patients with DM (5.6%) compared to those without DM (3.9%). By contrast, there was no major difference in the in-hospital mortality after femur fracture in male patients (8.0% with DM vs. 8.9% without DM). However, the differences displayed in Figure 2 are descriptive and univariable and do not take into account the impact of co-diagnoses.

### 3.3. Association Between Diabetes Mellitus and In-Hospital Mortality in Older People with Femur Fracture

A multivariable regression model, adjusted for co-diagnoses, found that diabetes mellitus was not significantly associated with in-hospital mortality among the total population of femur fracture patients (OR: 1.00; 95% CI: 0.84–1.20), irrespective of sex or age group (Table 2).

Table 3 displays the stepwise logistic regression. DM was associated with in-hospital mortality when adjusted for dementia, dyslipidemia, hypertension, and ischemic heart disease. After further adjustment for atrial fibrillation, the association between DM and in-hospital mortality initially became non-significant. When further adjusted for heart failure and CKD, however, the association reached an OR value of 1.00.

## 4. Discussion

In this large nationwide cross-sectional study of 12,880 hospitalized elderly patients with femur fractures, diabetes mellitus was only positively associated with in-hospital mortality in the age group over 90 years. Upon initial analysis, it seemed that DM patients had higher mortality rates. However, in a multivariable regression analysis, adjusting for co-diagnoses, we found that DM was not associated with in-hospital mortality. Furthermore, atrial fibrillation, heart failure, and CKD attenuated the effect of DM on in-hospital mortality.

In line with our findings, a previous study investigating DM as a risk factor for increased in-hospital mortality in elderly patients after proximal femur fracture found no association between DM and in-hospital mortality or even prolonged hospital stay [30]. Another multiple regression analysis investigating the 30-day mortality in patients with a femoral neck fracture ruled out the presence of DM as a risk factor but did identify male sex, increasing age, non-home source of admission, low hemoglobin levels, a history of myocardial infarction, concomitant chest infection on admission, increasing Charlson comorbidity score, and liver disease as risk factors for poorer mortality rates [7]. In both studies, the presence of comorbidities, age, sex, or other co-variables attenuated the effect of DM. This highlights the importance of accounting for confounding variables in medical research when interpreting results. Confounders are factors that can obscure the true relationship between the primary variable of interest and the outcome. In the present study, while diabetes alone was not found to be significantly associated with in-hospital mortality rates in femur fracture patients, the presence of additional age-related comorbidities such as CKD, atrial fibrillation, and heart failure influenced the analysis. This finding suggests that heart failure and CKD are critical factors that largely explain the mortality risk previously attributed to DM. Both heart failure and CKD are severe conditions that are commonly associated with worse clinical outcomes and higher mortality rates in hospitalized patients [31,32]. Therefore, once these conditions are accounted for, the independent effect of DM on mortality disappears, implying that DM’s apparent effect was likely mediated through its contribution to the development or exacerbation of these more severe comorbidities.

The relationship between mortality and comorbidities is both complex and multifaceted. Comorbidities diminish patients’ physiological reserves, heighten physiological stress, and compromise immune function, collectively increasing the risk of intra- or postoperative complications and prolonging hospital stays [33,34]. These factors, in turn, contribute to an increased risk of in-hospital mortality. Notably, extended hospitalization, which is often a consequence of rising complication rates, further exacerbates the risk of mortality following surgery [34]. It is therefore crucial to assess the impact of multiple coexisting conditions on in-hospital mortality risk. Consequently, recognizing the substantial impact of multiple coexisting conditions on in-hospital mortality risk is of great importance, particularly in elderly patients with diabetes mellitus—a prevalent condition within this demographic.

## 5. Limitations

It is important to acknowledge and address the limitations of this study. Firstly, the collection of data for patients with a diagnosis of femur fracture was based on the ICD-10 classification, and no further details were available regarding the fracture severity and type. Secondly, despite the inclusion and exclusion criteria applied to this dataset, other confounding variables may have influenced the results of this study (e.g., intra- or postoperative complications). Although the multivariable regression model was adjusted for various comorbidities, the authors acknowledge that it is possible that not all clinically relevant variables that might affect the outcome were addressed. Thirdly, the dataset is limited to information from German hospitals, which precludes the automatic generalization of the results to other countries. Fourthly, it is also important to note that our analysis was limited to mortality during the in-hospital stay and did not examine mortality rates in the period following discharge. In addition, the length of hospital stay varies between regions and countries. Patients with hip fracture had a mean stay of 4.2 days in Finland, 19.5 days in Ireland, and 17 days in Germany [35]. Fifthly, the database used contains information on surgeries performed during the hospital stay but not on the time at which the surgery took place. Therefore, no analysis could be conducted on the association between time to surgery and mortality. Sixthly, the average LOS was 17 days in our study. Mortality is usually correlated with the LOS and is higher in studies that focus on patients with longer hospital stays. This should be considered when interpreting our study results. Lastly, it should be noted that the data for this study were collected over an extended period (2013–2019), during which changes in hospital policies, guidelines, and practices may have influenced the results and trends. In particular, the impact of the global pandemic caused by the SARS-CoV-2 virus, which occurred within this timeframe, could have significantly impacted the mortality rates of the patients included. However, this study also has several strengths worth highlighting, particularly the large sample size and the extensive data collection from 36 hospitals across a number of German states.

## 6. Conclusions

In conclusion, this study demonstrates that DM alone does not constitute a significant risk factor for in-hospital mortality among older patients with femur fractures. However, the presence of comorbidities markedly influences the mortality rates in this patient population. Recognizing and understanding the complex interplay between DM and other chronic conditions will allow for targeted interventions to be developed for patients with femur fractures, potentially improving the outcomes. Our findings underscore the critical impact of chronic conditions on acute medical events. While the influence of secondary comorbidities on primary diseases is often underestimated, early identification of these factors can reveal underlying mechanisms that affect patient outcomes, facilitating more personalized treatment approaches. Accounting for these interactions can help minimize the risk of complications, leading to better prognoses for patients. Nevertheless, further research is essential to identify the specific risk factors contributing to increased mortality in femur fracture patients, independently of DM. The related insights will enable the development of more effective strategies to mitigate risks and enhance patient care.

## Figures and Tables

**Table 1:** Baseline characteristics of the study populations with and without diabetes mellitus (1:3 matching).

	Patients with Femur Fracture and Diabetes Mellitus (n = 3220)	Patients with Femur Fracture Without Diabetes Mellitus (n = 9660)	Standardized Mean Difference (SMD)
Age			−0.029
Mean age (SD)	83.0 (7.1)	83.2 (7.3)	
65–69 years, n (%)	159 (4.9)	493 (5.1)	
70–79 years, n (%)	738 (22.9)	2163 (22.4)	
80–89 years (%)	1760 (54.7)	5202 (53.9)	
≥90 years, n (%)	563 (17.5)	1802 (18.6)	
Sex, n (%)			0.000
Female	2225 (69.1)	6675 (69.1)	
Male	995 (30.9)	2985 (30.9)	
			*p*-value
Co-diagnoses, n (%)			
Dyslipidemia	855 (26.6)	1541 (16.0)	<0.001
Hypertension	2482 (77.1)	6255 (64.8)	<0.001
Chronic ischemic heart disease	707 (22.0)	1247 (12.9)	<0.001
Atrial fibrillation and flutter	964 (29.9)	2317 (24.0)	<0.001
Heart failure	715 (22.2)	1456 (15.1)	<0.001
Chronic kidney disease	939 (29.2)	1428 (14.8)	<0.001
All-cause dementia	814 (25.3)	2308 (23.9)	0.112

**Table 2:** Association between diabetes mellitus and in-hospital mortality in older patients hospitalized for femur fracture (multivariable logistic regression).

	Multivariable Logistic Regression *	
Subgroup of Patients	OR (95% CI)	*p*-Value
All patients	1.00 (0.84–1.20)	0.962
Patients aged 65–69 years	1.10 (0.28–4.22)	0.895
Patients aged 70–79 years	1.04 (0.65–1.68)	0.862
Patients aged 80–89 years	0.91 (0.70–1.17)	0.457
Patients aged ≥90 years	1.26 (0.94–1.66)	0.124
Female patients	1.23 (0.97–1.55)	0.085
Male patients	0.77 (0.59–1.02)	0.067

* Adjusted for co-diagnoses (dyslipidemia, hypertension, chronic ischemic heart disease, atrial fibrillation and flutter, heart failure, chronic kidney disease, and dementia).

**Table 3:** Association between diabetes mellitus and in-hospital mortality in older patients hospitalized for femur fracture (stepwise multivariable logistic regression).

Variables in Model	Stepwise Multivariable Regression	
	Association Between DM and In-Hospital Mortality OR (95% CI)	*p*-Value
DM only	1.19 (1.01–1.40)	0.043
DM + dementia	1.18 (1.01–1.40)	0.048
DM + dementia + dyslipidemia	1.24 (1.05–1.47)	0.013
DM + dementia + dyslipidemia + hypertension	1.27 (1.07–1.50)	0.014
DM + dementia + dyslipidemia + hypertension +ischemic heart disease	1.20 (1.01–1.43)	0.034
DM + dementia + dyslipidemia + hypertension + ischemic heart disease + atrial fibrilation	1.15 (0.97–1.37)	0.102
DM + dementia + dyslipidemia + hypertension + ischemic heart disease + atrial fibrilation + heart failure	1.07 (0.90–1.27)	0.455
DM + dementia + dyslipidemia + hypertension + ischemic heart disease + atrial fibrilation + heart failure + CKD	1.00 (0.84–1.20)	0.962

## Data Availability

The datasets used and analyzed during the current study are available from the corresponding author on reasonable request.
